# Arterial spin labelling MRI for brain tumour surveillance: do we really need cerebral blood flow maps?

**DOI:** 10.1007/s00330-023-10099-z

**Published:** 2023-08-11

**Authors:** Wouter H. T. Teunissen, Anna Lavrova, Martin van den Bent, Anouk van der Hoorn, Esther A. H. Warnert, Marion Smits

**Affiliations:** 1 Department of Radiology & Nuclear Medicine, Erasmus MC, Rotterdam, The Netherlands; 2 Brain Tumour Centre, Erasmus MC Cancer Institute, Rotterdam, The Netherlands; 3 Medical Delta, Delft, The Netherlands; 4 Department of Radiology, University of Michigan Hospital, Ann Arbor, MI USA; 5 Department of Neurology, Erasmus MC, Rotterdam, The Netherlands; 6 Medical Imaging Center, Department of Radiology, University Medical Center Groningen, Groningen, The Netherlands

**Keywords:** Brain, Brain neoplasms, Magnetic resonance imaging, Sensitivity and specificity

## Abstract

**Objectives:**

Arterial spin labelling (ASL) perfusion MRI is one of the available advanced MRI techniques for brain tumour surveillance. The first aim of this study was to investigate the correlation between quantitative cerebral blood flow (CBF) and non-quantitative perfusion weighted imaging (ASL-PWI) measurements. The second aim was to investigate the diagnostic accuracy of ASL-CBF and ASL-PWI measurements as well as visual assessment for identifying tumour progression.

**Methods:**

A consecutive cohort of patients who underwent 3-T MRI surveillance containing ASL for treated brain tumours was used. ROIs were drawn in representative parts of tumours in the ASL-CBF maps and copied to the ASL-PWI. ASL-CBF ratios and ASL-PWI ratios of the tumour ROI versus normal appearing white matter (NAWM) were correlated (Pearson correlation) and AUCs were calculated to assess diagnostic accuracy. Additionally, lesions were visually classified as hypointense, isointense, or hyperintense. We calculated accuracy at two thresholds: low threshold (between hypointense-isointense) and high threshold (between isointense-hyperintense).

**Results:**

A total of 173 lesions, both enhancing and non-enhancing, measured in 115 patients (93 glioma, 16 metastasis, and 6 lymphoma) showed a very high correlation of 0.96 (95% CI: 0.88–0.99) between ASL-CBF ratios and ASL-PWI ratios. AUC was 0.76 (95%CI: 0.65–0.88) for ASL-CBF ratios and 0.72 (95%CI: 0.58–0.85) for ASL-PWI ratios. Diagnostic accuracy of visual assessment for enhancing lesions was 0.72.

**Conclusion:**

ASL-PWI ratios and ASL-CBF ratios showed a high correlation and comparable AUCs; therefore, quantification of ASL-CBF could be omitted in these patients. Visual classification had comparable diagnostic accuracy to the ASL-PWI or ASL-CBF ratios.

**Clinical relevance statement:**

This study shows that CBF quantification of ASL perfusion MRI could be omitted for brain tumour surveillance and that visual assessment provides the same diagnostic accuracy. This greatly reduces the complexity of the use of ASL in routine clinical practice.

**Key Points:**

*• Arterial spin labelling MRI for clinical brain tumour surveillance is undervalued and underinvestigated.*

*• Non-quantitative and quantitative arterial spin labelling assessments show high correlation and comparable diagnostic accuracy.*

*• Quantification of arterial spin labelling MRI could be omitted to improve daily clinical workflow.*

## Introduction

The management of patients with a brain tumour is hampered by various diagnostic challenges. One of these is the differentiation between tumour progression and treatment-related abnormalities [1]. Conventional MRI techniques are not able to reliably differentiate between these two entities, both displaying an increase in contrast enhancement and/or T2-hyperintensity in the treated tumour region [2]. Perfusion MRI visualises the perfusion of blood within a tissue, such as in tumour. The general idea is that an increased perfusion compared to healthy tissue is associated with tumour progression due to neovascularisation, while a decreased perfusion is seen in treatment-related abnormalities due to inflammatory and/or thrombo-embolic tissue changes [3]. We do not address the phenomenon pseudo-regression, as this occurs in relation to anti-angiogenetic medication which is not standard of care in the Netherlands for glioma treatment.

Different perfusion MRI techniques are available for clinical use: dynamic contrast-enhanced (DCE), dynamic susceptibility contrast (DSC), and arterial spin labelling (ASL) MRI. While DSC is by far the most commonly used [4], ASL has several advantages over DSC. ASL uses magnetically labelled blood to create contrast instead of an exogenous paramagnetic contrast agent, thus being entirely non-invasive, and ASL is commonly implemented with an imaging read-out that is less sensitive to susceptibility artefacts than commonly used read-outs in DSC [5, 6]. The technique can therefore be used after haemorrhage, around the skull base, and in the presence of metallic surgical material. Importantly, ASL perfusion measurements are based on labelled, endogenous blood water which acts as a freely diffusible tracer and therefore do not suffer from blood brain barrier leakage effects that affect DSC perfusion measurements. The main disadvantages of ASL are its lower signal to noise ratio and longer scanning time than DSC [7]. Also, there is still considerable unfamiliarity with the technique, which is in part due to its perceived complexity of post-processing and interpretation.

The acquisition of ASL is based on a subtraction of the values from two differently acquired MRI series. The first series is a control series of the area of interest. The second series is acquired after magnetically labelling the inflowing arterial blood (usually at the cervical level). Subtraction of the labelled from the control series provides the raw perfusion-weighted image (ASL-PWI) in which every voxel has a unitless, arbitrary value proportional to the amount of labelled blood that has reached this voxel [8]. A kinetic model can be used to quantify ASL-PWI to generate cerebral blood flow (CBF) perfusion maps in which each voxel represents perfusion with a specific unit, i.e. mL/100 g/min [9].

In daily clinical practice, CBF quantification is an extra step during post-processing of the perfusion data, which interferes with the radiologist’s or radiographer’s workflow and may even be costly in case post-processing tools need to be additionally acquired. While quantification of perfusion is a commonly stated advantage of ASL over other perfusion MRI techniques, the question is whether it is really needed for routine clinical use.

In non-quantitative techniques such as DSC, it is usual to calculate a ratio between the neoplastic lesion and the normal appearing white matter (NAWM) [10]. A region of interest (ROI) is placed in the lesion and the mean value is divided by the mean value of the same ROI in the NAWM. The result of this semiquantitative approach is a unitless value which can be compared within and across patients. Another possible approach is a visual assessment (‘eyeballing’) of areas of relative hyperperfusion, by comparing to the signal intensity of healthy cortex [11]. Both approaches are appealing for clinical practice, due to a more efficient workflow.

The aim of this study was to evaluate how semiquantitative as well as visual assessment of ASL derived ASL-PWI maps compare to the use of quantitative ASL-CBF maps in patients with a variety of enhancing and non-enhancing treated brain tumours. We hypothesise that ASL-PWI and ASL-CBF ratios are highly correlated, thus obviating the need for quantification of CBF and thereby vastly simplifying the use of ASL perfusion in the routine clinical setting of brain tumour surveillance.

## Methods

### Patient selection

In this single-centre retrospective study, a cohort of consecutive patients was included, who were scanned for surveillance of any intra-axial brain tumour between 1 January and 31 December 2019 at 3-T MRI at the Erasmus MC, Rotterdam, the Netherlands. Clinical information was obtained from the electronic health records, and consisted of information on general demographics, clinical diagnosis, and histopathology (according to the WHO 2016 classification [12]). Follow-up (for a minimum of 3 months) data were used to confirm clinical diagnosis in case no histopathology was available. Both radiological and clinical information were used to define tumour progression, pseudoprogression (i.e. treatment-related abnormalities), or stable disease, in line with the criteria formulated by Ellingson et al [13]. This study design was reviewed by the Erasmus MC Medical Ethics Committee (MEC-2020–0267) and performed according to the declaration of Helsinki and the Dutch regulations on medical research.

### MRI protocol

MRI scans were performed on two 3T MRI scanners (GE Healthcare) using a 32- or 48-channel head coil. ASL was acquired as a 3D pseudocontinuous (PCASL) sequence with spiral readout and background suppression using flip angle (FA) = 111°, echo time (TE) = 10.6 ms, repetition time (TR) = 4635 ms, label duration of 4 s, and a single post-labelling delay (PLD) of 1.5 s; the reconstructed voxel size was 1.9 × 1.9 × 3.5 mm^3^. No vascular crushing was used during acquisition. This sequence is the available product sequence from this vendor.

ASL-PWI maps consisted of the averaged subtraction of the unlabelled minus the labelled acquisitions as provided directly by the scanner without any additional post-processing. The ASL-PWI maps are not quantitative, containing arbitrary pixel values.

ASL-CBF maps were calculated with a vendor-specific software package (AW Server, GE Healthcare) and in line with the ASL white paper recommendations [14]. This software package uses a quantification model as described by Maleki et al [15] to calculate CBF in mL/100 g/min; these maps are thus referred to as quantitative maps.

### Image analysis

**Fig. 1:**
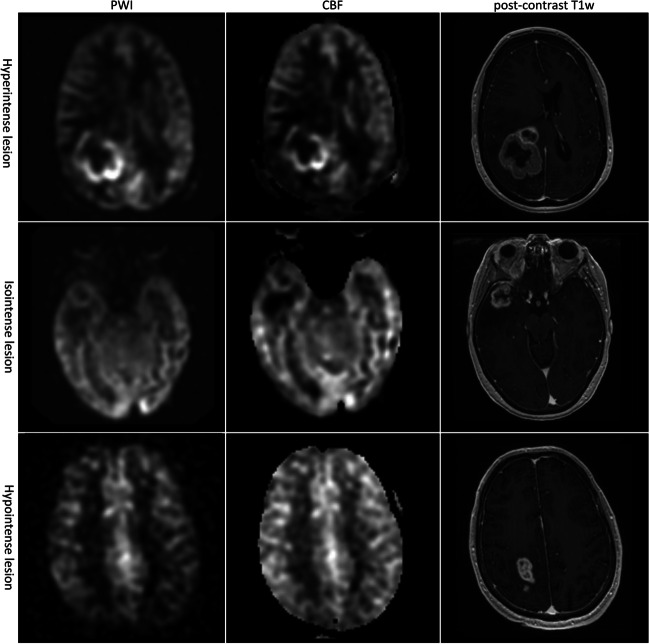
Examples of hyper-, iso-, and hypointense enhancing lesions (as determined on post-contrast T1w imaging) on ASL-derived ASL-PWI and ASL-CBF maps

After visual quality assessment, mainly focusing on motion artefacts and tissue contrast [16], first a quantitative approach was taken. Lesions were identified on post-contrast T1w images or on T2w/T2w-FLAIR images if contrast enhancement was absent. Measurements were done in Radiant DICOM Viewer by placing an oval or circular region of interest (ROI) of approximately 70 mm^2^ (mean 78 ± 14 mm^2^) in a representative part of the lesion (which had at minimum the size of the ROI) with the highest perfusion (‘hot spot’) as visually determined on the ASL-CBF map, and copied to the contralateral normal appearing white matter (NAWM) on the same image slice [17]. Window level was also chosen visually, optimising the differentiation of grey and white matter. For anatomical reference, overlays of the perfusion map on the post-contrast T1w image were used, especially to ensure inclusion of the tumour and exclusion of vessels. In all cases, a grey-scale was used. The ROIs were then copied to the ASL-PWI map, such that the measurements of ASL-CBF and ASL-PWI within each lesion were derived from identically sized and placed ROIs. Ratios between the tumour and NAWM were calculated by dividing the mean tumour ROI value by the mean NAWM ROI value. All quantitative ROI measurements were done by AL and verified by WT. Secondly, the lesions’ ASL-PWI signal intensities were classified by two raters (W.T., neuroradiologist in training, 4 years of experience and A.H., neuroradiologist, 9 years of experience) as hypointense, isointense, or hyperintense compared to healthy appearing cortex (Fig. 1). Both were blinded to the tumour histopathology and follow-up data.

### Data analyses

Statistical analyses were performed using RStudio. Pearson’s correlation coefficient and 95% confidence intervals (CI) were calculated between the ASL-CBF ratios and ASL-PWI ratios for each lesion. A cluster bootstrapping approach was used to correct for potential dependency of multiple lesions within the same patient. To assess the relationship between ASL-CBF ratios and visual assessment, boxplots were created.

Correlations were determined in all patients combined, as well as in subgroups of patients with enhancing lesions, enhancing and non-enhancing glioma, brain metastasis, and primary central nervous system lymphoma (PCNSL). The initial diagnosis was histopathologically proven. The diagnosis at the moment of scanning for surveillance (PD of PsP) was determined radiologically or histopathologically. Diagnostic accuracy of determining tumour progression was assessed as the area under the receiver operating characteristics (ROC) curves (AUC) for ASL-CBF ratios and ASL-PWI ratios. This was only done in the subgroup of patients with enhancing lesions, as it is primarily in enhancing lesions that the differentiation between tumour progression and treatment-related abnormalities may be problematic. The diagnostic accuracy based on visual assessment of the ASL-PWI maps was assessed by calculating sensitivity, specificity, and the proportion of correctly classified patients with two different thresholds. The first threshold, the low threshold, was between hypointense and isointense (so both isointense and hyperintense signals are considered ‘test positive’). The second threshold, the high threshold, was between isointense and hyperintense (so only hyperintense signal is considered ‘test positive’). ASL-CBF ratios of the three different visual categories were compared using a Kruskal–Wallis test. The interobserver agreement (Cohen’s Kappa) was calculated for both thresholds. In cases of disagreement, a third reader (M.S., neuroradiologist, 18 years of experience) performed an adjudication to obtain a final assessment.

## Results

### Patient and lesion characteristics

A total of 122 patients with a total of 188 lesions were evaluated. Fifteen lesions (8%) were excluded from further analysis due to severe motion artefacts (*N* = 10) or being too small to measure (*N* = 5). A total of 173 lesions measured in 115 patients were deemed eligible for further analysis. Table 1 shows the patient characteristics. All patients had undergone treatment (radiation and/or chemotherapy). Final diagnosis was determined in 10 patients by histopathology, in all other patients by imaging. Identified lesions were 80 enhancing glioma, 52 non-enhancing glioma, 31 enhancing metastases, and 10 residual lesions after treated PCNSL.

**Table 1:** Patient characteristics

*Glioma*	
No. of patients	93
No. of lesions	132
Age in years (mean ± SD)	52.9 ± 12.8
Gender (male/female)	64/29
Enhancing lesions	80
Non-enhancing lesions	52
Glioma subtypes:	
Diffuse astrocytoma, IDH-mutant	6
Diffuse astrocytoma, IDH-wildtype	1
Diffuse astrocytoma, NOS	7
Oligodendroglioma, IDH-mutant and 1p/19q-codeleted	14
Oligodendroglioma, NOS	5
Oligoastrocytoma, NOS	2
Anaplastic astrocytoma, IDH-mutant	6
Anaplastic astrocytoma, IDH-wildtype	1
Anaplastic astrocytoma, NOS	4
Anaplastic oligodendroglioma, IDH-mutant and 1p/19q codeleted	2
Anaplastic oligodendroglioma, NOS	1
Glioblastoma, IDH-wildtype	20
Glioblastoma IDH-mutant	5
Glioblastoma, NOS	8
Primary brain tumour NOS†	11
*Metastasis*	
No. of patients	16
No. of lesions	31
Age in years (mean ± SD)	57.1 ± 12.8
Gender (male/female)	5/11
Enhancing lesions	31
Non-enhancing lesions	0
Primary tumour:	
Lung cancer	7
Breast cancer	3
Melanoma	2
Other	4
*PCNSL*	
No. of patients	6
No. of lesions	10
Age in years (mean ± SD)	61.8 ± 13.4
Gender (male/female)	4/2
Enhancing lesions	8
Non-enhancing lesions	2

*PCNSL* primary central nervous system lymphoma

^†^ Diagnosis according to the WHO 2016 classification could not be retrieved

### Relation between ASL-CBF ratios, ASL-PWI ratios, and visual classification

**Fig. 2:**
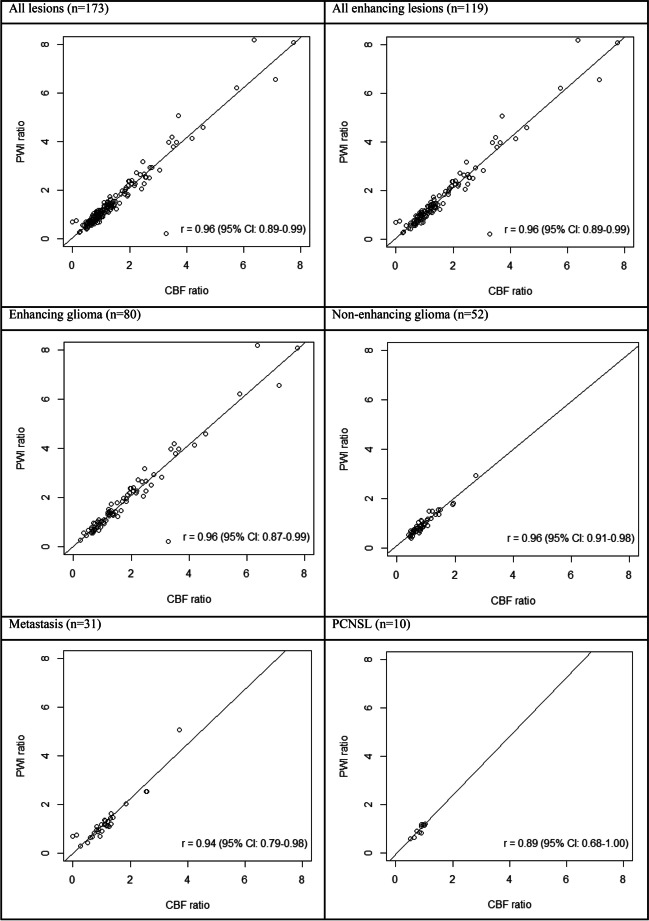
Correlation plots and coefficients (*r*) between ASL-derived ASL-CBF and ASL-PWI ratios (lesion level)

Pearson’s correlation coefficients between ASL-derived ASL-CBF ratios and ASL-PWI ratios were calculated for all lesions together and for subsets consisting of all enhancing lesions, enhancing and non-enhancing glioma, metastasis, and PCNSL (Fig. 2). We found a very high correlation between ASL-CBF ratios and ASL-PWI ratios of 0.96 (95% CI: 0.89–0.99) for all lesions combined. Subsets of enhancing lesions only, enhancing glioma, and non-enhancing glioma also each showed a correlation coefficient of 0.96 (95% CI: 0.89–0.99, 0.87–0.99, and 0.91–0.98 respectively). The subset of metastasis showed a correlation coefficient of 0.94 (95% CI: 0.79–0.98) and that of PCNSL 0.89 (95% CI: 0.68–1.00), the latter subset only consisting of 10 lesions.

**Fig. 3:**
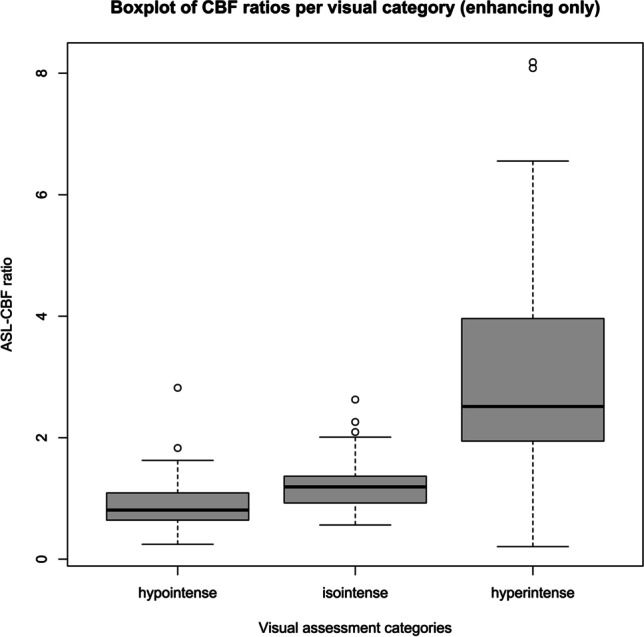
Boxplots of ASL-CBF ratio values per visual category

Visual assessment of lesion intensity by two different readers (W.T. and A.H.) showed an interobserver agreement for the low threshold of 75% (Cohen’s Kappa = 0.48 (95% CI: 0.28–0.69)) and 89% (Kappa = 0.74 (95% CI: 0.59–0.91)) for the high threshold. Figure 3 shows the boxplots of the three visual categories (hypointense, isointense, and hyperintense) with their corresponding ASL-CBF ratios. Hypointense lesions showed a mean ASL-CBF ratio of 0.86 (SD: 0.37), isointense lesions a mean ASL-CBF ratio of 1.24 (SD: 0.44), and hyperintense lesions a mean ASL-CBF ratio of 3.00 (SD: 1.80). ASL-CBF ratios of the hyperintense category showed a significant difference (*p* < 0.001) with both the isointense and the hypointense categories.

### Diagnostic accuracy

**Fig. 4:**
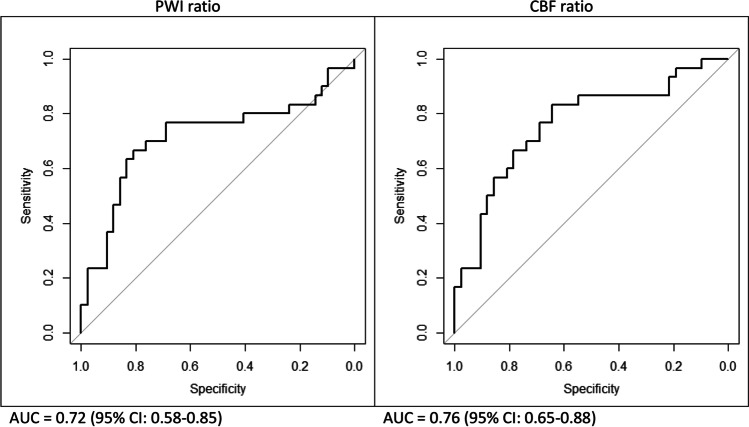
ROC curves for determining tumour progression with the ASL-PWI ratio and the ASL-CBF ratio

The AUC as a measure of diagnostic accuracy of determining tumour progression with the quantitative approach was 0.76 (95% CI: 0.65–0.88) when based on ASL-CBF ratios, and 0.72 (95% CI: 0.58–0.85) when based on ASL-PWI ratios (Fig. 4).

After adjudication (M.S.), the diagnostic accuracy of determining tumour progression using the visual assessment showed a sensitivity of 0.83 and a specificity of 0.64 for the low threshold, and a sensitivity of 0.57 and a specificity of 0.83 for the high threshold (Table 2). The diagnostic accuracy in terms of the proportion of correctly classified patients (true positives + true negatives) out of the total number of patients was 0.72 both for the low and the high thresholds.

**Table 2:** Diagnostic accuracy of determining tumour progression using visual assessment in patients with enhancing lesions only (*N* = 72 patients)

	Low threshold*	High threshold*
Sensitivity	0.83	0.57
Specificity	0.64	0.83
Diagnostic accuracy	0.72	0.72

^*^Low threshold: threshold is between hypointense and isointense signal intensity on perfusion weighted imaging (ASL-PWI). High threshold: threshold is between isointense and hyperintense signal intensity on ASL-PWI

## Discussion

In this retrospective study, we investigated the correlation between quantitative ASL-CBF maps and non-quantitated ASL-PWI maps in order to assess whether CBF quantification could be omitted in the context of brain tumour surveillance with a particular focus on enhancing lesions. Our second aim was to investigate the diagnostic accuracy for determining tumour progression of ASL-CBF and ASL-PWI measurements as well as simple visual assessment (‘eyeballing’) of ASL-PWI maps. We found a very high correlation (0.96) and comparable AUCs (0.72–0.76) of ASL-CBF ratios and ASL-PWI ratios, indicating that a ASL-PWI ratio provides the same clinical information as a ASL-CBF ratio. We also found a comparable diagnostic accuracy of visual assessment (0.72, both at high and low thresholds). These findings indicate that each of the tested approaches results in comparable diagnostic accuracy, hence either approach could be applied.

Working with perfusion ratios, the lesion value divided by the NAWM value, has been used for DSC MRI perfusion for a long time and provides a unitless value, i.e. rCBV. This method is also currently the most common ASL method to quantify the perfusion in an area of interest, as described in a meta-analysis by Wang et al [18]. Not only the method of using ratios is similar, also the diagnostic accuracy is comparable. A meta-analysis by van Dijken et al [19] reported a pooled diagnostic accuracy for DSC perfusion of 18 studies using CBV ratios with a sensitivity of 87% and a specificity of 86%. Although this is slightly higher than reported here in this study, it is in line with the diagnostic accuracy we have found. Also a comparison of ASL and DSC previously reported by this group provides a comparable diagnostic accuracy of ASL (AUC = 0.73) and DSC (AUC = 0.78) [20].

Using a ASL-PWI ratio instead of a ASL-CBF ratio could improve the workflow in daily practice because the quantification step could be omitted. As stated in the “Introduction”, there is still considerable unfamiliarity with routine usage of ASL, especially because of the perceived complexity of post-processing the imaging data and interpretation of results. Our findings indicate that both methods, with and without the usage of additional post processing and interpretation, provide the same clinical information. Of course, the amount of time saved by using this method differs per software package. To the best of our knowledge, no other studies are available using PCASL (implemented according to the most recent recommendations [14]), to investigate the perfusion of brain tumours with either an ASL-PWI approach or the comparison between ASL-PWI and ASL-CBF.

As expected, we found a very high correlation between the ASL-derived (non-quantitative) ASL-PWI ratios and the quantitative ASL-CBF ratios. After all, both maps are derived from the same source data while the use of ratios—through internal normalisation to the patient’s own reference values—removes dependency on absolute quantification. Although this correlation was very high (0.89–0.96), it is not perfect. This small mismatch could be explained by the model used for CBF quantification. The kinetic model used for CBF quantification relies on certain assumptions of underlying cerebrovascular physiology which could be influenced by the pathologies of interest here. Particular in pathology, violations of these underlying assumptions can result in inaccuracies of quantification [9, 15] and some potential violations may be applicable to our imaging data. For instance, the T1 decay of tumour tissue is different from healthy brain tissue [21], causing inaccuracies in CBF quantification, which may partly explain the small difference in correlation coefficient. Also, the arterial transit time can be altered in tumour tissue [14], which could further contribute to the small mismatch between ASL-PWI and ASL-CBF in the current single PLD PCASL approach. Note that this could be more appropriately accounted for by using multi- rather than single PLD ASL. We found a slightly lower correlation of 0.89 for the PCNSL subgroup (6 patients, 10 lesions), suggesting stronger violation of the assumptions in the kinetic model in this specific pathology. This is worth investigating in a lager sample size, as our subset of PCNSL patients was very small (*n* = 6). In line with the very high correlation between ASL-CBF and ASL-PWI, the AUC is also comparable, due to the fact that the AUC is directly related to the measured perfusion in relation to the underlying pathology.

Although quantification could be useful in specific cases, visual assessment (‘eyeballing’) without performing any measurements, would make the workflow even more efficient. This study shows that when a lesion is hyperintense (compared to the normal cortex), the average ASL-CBF ratio could be expected to be around 3 (3.00 ± 1.80), which is associated with tumour progression as previously described by Seeger et al [22]. This is in line with previously published data on the comparison between ASL-derived CBF and DSC-derived rCBV [20]. Diagnostic performance to determine tumour progression using visual assessment showed moderate diagnostic accuracy of 0.72, for both thresholds. In line with previous work [11], the interobserver agreement for this method was substantial [23] when the high threshold was used. At the low threshold, i.e. differentiating between iso- and hypo-intense ASL-PWI signals, this agreement was lower but still moderate. This could indicate that although the diagnostic accuracies of the high and the low thresholds are the same, there is a preference for the high threshold because the much higher interobserver agreement makes it a more reproducible method. Even so, although this may be a very efficient method, visual assessment is rater-dependent, and it is difficult to measure differences over time within one patient.

Some limitations apply to this study. While we included a large dataset of 115 patients and a total of 173 lesions, this is a retrospective cohort study. In most cases, histopathological confirmation was not available; therefore, the follow-up diagnosis was made radiologically and clinically, taking the course of symptoms and imaging abnormalities over time into account. Even though heterogeneity within a lesion could occur, we did not do an intra-tumoural analysis. We focused on the region of highest intensity within the lesion because this hot-spot approach is consistent with current clinical practice. Although reliable and reproducible information is required during follow-up, that is not always possible in a routine clinical setting and available for retrospective analysis [24].

All patients described in this study were scanned at 3 T; thus, applicability at 1.5 T was not investigated. Only one vendor (GE) and one sequence is  used. It is important to realise that there are differences between vendors and sequences; therefore, these results are not necessarily applicable to other situations than described in this study. For future research, it would be good to repeat this study in a multicentre and multivendor design, preferably prospectively. It is also important to emphasise that these results are from patients with brain tumours. For other ASL indications such as cerebrovascular disease or neurodegenerative disease, findings could be different, and these pathologies were not investigated in this study. It is also important to mention that a grey-scale approach was used for all ASL-CBF and ASL-PWI maps and no colour coding scheme was used. This is important for implementation of these results as many colour schemes are not perceptual linear which might alter the interpretation [25].

In conclusion, this study demonstrates that ASL-PWI ratios and ASL-CBF ratios are highly correlated and that both these approaches as well as a simple visual assessment all show comparable diagnostic accuracy, suggesting that ASL-CBF quantification could be omitted in patients with brain tumour surveillance at 3 T.

## Abbreviations

ASL: Arterial spin labelling
ASL-PWI: Perfusion weighted imaging
AUC: Area under the curve
CBF: Cerebral blood flow
DCE: Dynamic contrast enhanced
DSC: Dynamic susceptibility contrast
MRI: Magnetic resonance imaging
NAWM: Normal appearing white matter
PCASL: Pseudocontinuous ASL
PCNSL: Primary central nervous system lymphoma
PLD: Post-labeling delay
ROC: Receiver operating characteristics
ROI: Region of interest

## Declarations

### Guarantor

The scientific guarantor of this publication is Prof. Dr. Marion Smits.

### Statistics and biometry

No complex statistical methods were necessary for this paper.

### Study subjects or cohorts overlap

Some study subjects or cohorts have been previously reported in Lavrova et al 2022 (https://doi.org/10.3389/fonc.2022.849657).

### Methodology

• Retrospective

• Cohort

• Performed at one institution
